# Clinic-epidemiological analysis of an Otorhinolaryngology Emergency Unit Care in a Tertiary Hospital

**DOI:** 10.1590/S1808-86942011000400004

**Published:** 2015-10-19

**Authors:** Paula Lobo Furtado, Marcio Nakanishi, Gustavo Lara Rezende, Ronaldo Campos Granjeiro, Taciana Sarmento de Oliveira

**Affiliations:** 1 Medical otorhinolaryngologist at the Hospital de Base, Federal District; 2 Doctoral degree in otorhinolaryngologist, São Paulo University. Medical doctor, Brasilia University and Hospital de Base, Brasília. Volunteer professor, Medical School, Brasília University; 3 Medical doctor, master's degree student, Brasília University; 4 Doctoral student, Brasília University. Medical otorhinolaryngologist, Hospital de Base, Federal District; 5 Medical otorhinolaryngologist. Head of the Otorhinolaryngology Unit, Hospital de Base, Federal District

**Keywords:** emergency service, hospital, epidemiology, otolaryngology

## Abstract

**Abstract:**

Emergencies are common in our Otorhinolaringology specialty. However, the clinical and epidemiological features are not very well known.

**Objectives:**

To evaluate the clinical and epidemiological profiles of otorhinolaryngological disorders in an emergency unit of a tertiary hospital, and to determine the appropriateness of the level of health care for a tertiary hospital.

**Materials and methods:**

An analytical study using data records of an otorhinolaryngological emergency unit at a tertiary hospital in the Federal District for a year, full time, and no screening. The age, sex, arrival time and clinical diagnosis were evaluated. The entities were separated into cases of pharingolaryngoesthomatology, otology, rhinology, and head and neck surgery. These were evaluated according to the urgency level, the required care, and the arrival time.

**Results:**

26,584 data records were selected, of which 2,001 were excluded. The group comprised 54.48% women, and 45.51% men. Otological complaints (62.27%) prevailed. 61.26% of cases were considered emergencies. Only 9.7% of those required medium or high complex resources for resolution.

**Conclusion:**

The study showed that 61.26% of the otorhinolaryngological cases are emergencies, and only 9.7% required medium or high complexity resources.

## INTRODUCTION

Clinical otorhinolaryngology includes emergency care that generally takes place in secondary or tertiary care hospitals.^1^ Access to these services may be open or by referral. At present the number of patients seen in emergency units has gradually increased^1^, ^2^, ^3^. The subjective nature of the concept of urgency interferes with adequate care in many open access services^4,^^5^, as non-urgent cases tend to overcrowd emergency units^4,^^6,^^7^. The concept of urgency may vary; it depends on social, familiar, work-related, bureaucratic, sanitary, patient-related, and medical situations^2^.

Few studies have been published on the characteristics of otorhinolaryngological diseases seen at emergency units^2^, especially the severity of cases and the appropriateness of the level of care in institutions that provide emergency care. Timsit et al.^3^ have reported that only 10% of visits to emergency units are truly urgent cases.

Rivero et al.^7^ published data gathered at an otorhinolaryngology emergency unit, and concluded that it is essential to define the truly urgent cases for adequate planning and care to be instituted. These authors also concluded that less than 1/3 of visits could be considered as truly urgent cases.

The public hospital network in Brasilia (Federal District or DF) has a tertiary level hospital and 17 regional secondary level hospitals, which serve an estimated 2,455,903 inhabitants^8^, and are referrals for other states such as Goiás, Minas Gerais, and Bahia. This hospital is the only public hospital that has full time walk-in access emergency otorhinolaryngological care.

The purposes of this study were to evaluate the clinical and epidemiologic features of otorhinolaryngological diseases of patients seen at an emergency unit of a tertiary hospital in the Federal District, and to assess the appropriateness of the level of care relative to the care provided by this hospital, according to the hierarchical principle set in the Law 8080/90 of the Federal Constitution, which describes the organization of healthcare services and that instituted the unified health system (Sistema Único de Saúde or SUS).

## MATERIAL AND METHODS

An analytical cross-sectional study was carried out from October 2007 to 2008 at an otorhinolaryngology emergency unit. Data were gathered from patient registries, and consisted of the following items: age, sex, hour, and clinical diagnosis. The clinical diagnosis, which was used for classifying the cases, was based on the main complaint of patients.

The otorhinolaryngologist on duty and medical residents provided full time healthcare. Patients were not screened, so that every person seeking emergency care for otorhinolaryngological complaints was seen. All patients seen at the otorhinolaryngology emergency unit were enrolled in this study. Patients were excluded based on the hospital admittance reports, on non-otorhinolaryngological diseases, return visits, and incomplete files.

After the clinical diagnosis, patients were subdivided into otology, rhinology, pharyngolaryngostomatology, and head & neck surgery cases. The hospital admittance rate was measured by relating the number of cases that stayed in hospital for more than 24 hours with the total number of cases.

Also based on the diagnosis, etiology, and pathology, events were divided into urgent and non-urgent cases. All diagnoses listed on Frame 1 were defined as urgencies.

**Frame 1:**
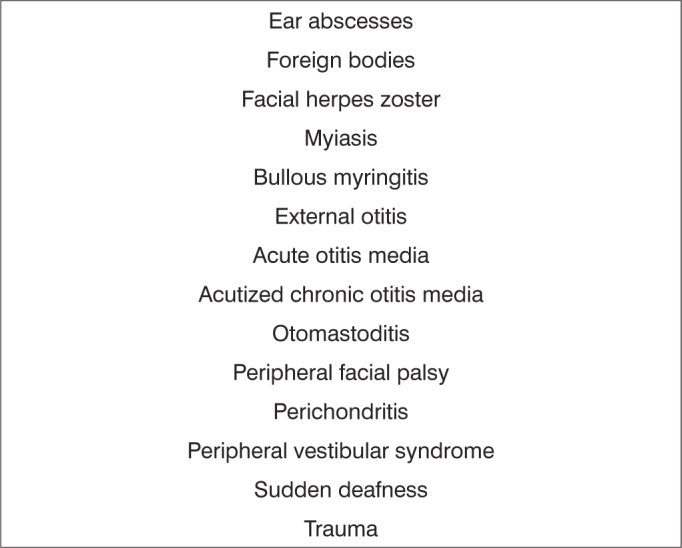
List of diagnostic hypotheses in cases considered as urgent - otology

**Frame 2:**
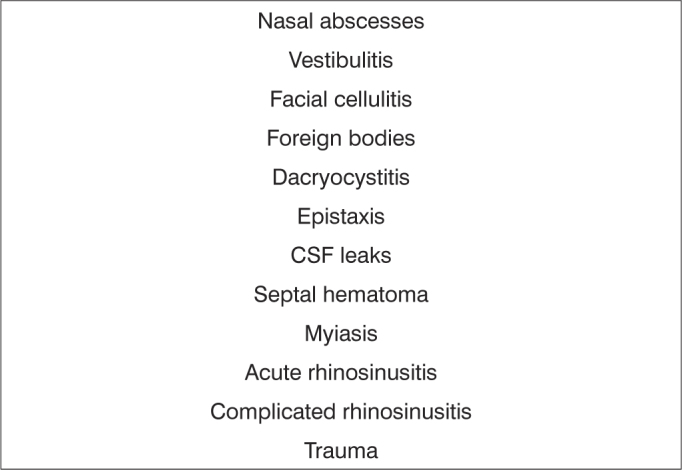
List of diagnostic hypotheses in cases considered as urgent - rhinology

**Frame 3:**
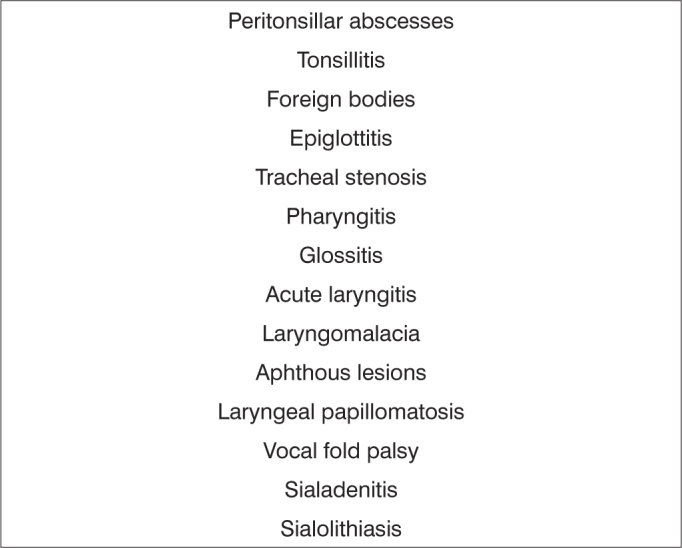
List of diagnostic hypotheses in cases considered as urgent - pharyngolaryngostomatology.

**Frame 4:**
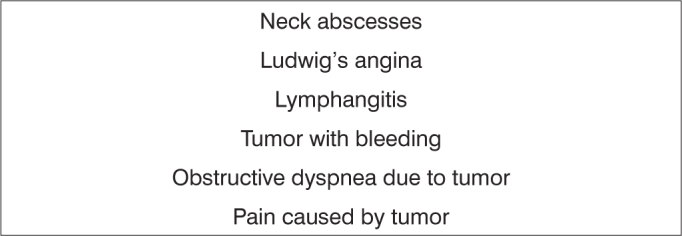
List of diagnostic hypotheses in cases considered as urgent - head & neck surgery.

Urgent cases were subdivided into tertiary urgencies - cases that required medium or high complexity diagnostic and/or therapeutic methods and equipment (computed tomography, magnetic resonance imaging, audiological testing, videolaryngoscopy, nasofibroscopy, bronchofibroscopy, electroneurography, surgical endoscopes or microscopes) - and non-tertiary urgencies, which did not require these resources.

Urgent cases were allocated into subgroups according to criteria described by Cuchi^9^ (1989): inflammatory/infectious events, trauma, bleeding, foreign bodies, tumors, functional conditions, sensorineural diseases, respiratory diseases, and non-classified conditions.

Cases were also subdivided into three groups according to the hour of the day: a daytime group (patients seen from 7 a.m. to 7 p.m.; a night group (patients seen from 7 p.m. to midnight); and a late night group (patients seen from midnight to 7 a.m.).

The SPSS version 13.0 statistical software was used for data analysis. Categories were described in numbers (n) and percentages (%). The institutional review board of the health Office of the Federal District authorized this study (research project no. 091/08).

## RESULTS

From 1 October 2007 to 30 September 2008, 26,584 were seen at the otorhinolaryngology emergency unit of a tertiary hospital. There were 2,001 files excluded for the following reasons: non-otorhinolaryngological conditions - 960 cases (48%); return visits - 481 cases (24%); and 560 files with incomplete data (28%). Of the remaining 24,583 files, 13,393 (54.48%) were of female patients, and 11,190 (45.51%) were of male patients (a 1.19 female to male ratio). The age groups were 0 to 15 years (22.71% of cases), 16 to 65 years (71.96%), and over 66 years (5.31%).

Subspecialties were as follows: 15.309 otologic complaints (62.27%); 4,561 rhinologic complaints (18.55%); 4,203 pharyngolaryngostomatologic complaints (17.09%); and 510 head & neck surgery cases (2.07%) (Table 1).

**Table 1:** Absolute number and percentage of visits to the otorhinolaryngology emergency unit at the Hospital de Base, Federal District, by subspecialty.

Subspecialty	Total	Percentage
Otology	15,309	62.27
Rhinology	4,561	18.55
Pharyngolaryngostomatology	4,203	17.09
Head & neck surgery	510	2.07
Excluded registries	2,001	7.05
Total	26,584	100

Among 24,583 otorhinolaryngology related events, 15,060 (61.26%) were considered urgent, and 9,523 (38.73%) were considered non-urgent cases. Frame 1, Frame 2, Frame 3, Frame 4 present the diagnostic hypotheses of the urgent cases. There were 580 hospital admittances in which patients remained in hospital for over 24 hours (hospital admittance rate - 2.35%).

Among the urgencies, 12,674 cases (51.55%) were considered non-tertiary, and 2,386 cases (9.7%) were events that required middle and high complexity measures - tertiary urgencies.

Urgencies were subdivided by etiology based on Cuchi's^9^ criteria, as shown on Table 2.

**Table 2:** Etiology of cases considered as urgent, subdivided according to Cuchi's^9^ criteria.

Subdivided	Total number of urgencies	Percentage
Inflammation/Infection	9,897	65.71
Foreign bodies	2,526	16.77
Trauma	1,190	7.9
Hemorrhage	786	5.21
Sensorineural disorders	344	2.28
Tumors	249	1.65
Respiratory disorders	16	0.10
Functional disorders	00	00
Not classified	00	00
Total	15,060	100

Table 3 shows the distribution of cases according to the time of day and weekdays in which patients were seen.

**Table 3:** Absolute number and percentage of visits to the otorhinolaryngology emergency

	Absolute number	Percentage
Daytime group	19,370	78.79
Night group	3,886	15.80
Late night group	1,327	5.39
Total	24,583	100

## DISCUSSION

Urgent otorhinolaryngology cases comprise an important portion of emergency events in major urban centers^10^; hospital services in these areas are often overcrowded because of high patient demand. This situation becomes a public health issue because it worsens the level of care that is provided to all cases. About 960 events were of disease unrelated to otorhinolaryngology, and 9,523 cases were not otorhinolaryngological urgencies. These cases overcrowd emergency units, reduce the quality of care to truly urgent cases, increase the cost of healthcare, and reduce the efficiency of healthcare services.

Few published papers have described the reality in otorhinolaryngology emergency units^1,^^9,^^11,^^12^. Emergency units have become an alternative to a repressed demand for specialists in outpatient clinics^11^; many patients seek these services for the treatment of diseases that could be resolved at an outpatient level. Rivero et al.^11^ found that 35% to 40% of cases in an otorhinolaryngology emergency unit were justifiable urgencies. Sanches-Alcon et al.^10^ found a 56% rate. This percentage is even lower in other papers: Timsit et al.^6^ concluded that only 10% of cases were truly urgent.

Of 24,583 cases, 15,060 (61.26%) were considered urgent. This percentage is higher than other published reports in the literature^6,^^10,^^11^. We believe that one of the reasons for this higher percentage of urgent cases was the use of more widely defined criteria compared to those in the literature. Furthermore, we consider that such comparisons are poorly valid, because those few studies adopt different criteria for urgencies and studied different populations. Standardization of epidemiologic studies of urgencies is needed in the literature, as the papers we found present data in non-standard formats, which precludes comparisons. An additional factor is the lack of another otorhinolaryngology emergency unit in the Federal District; thus, all emergency cases are seen at our institution, which results in a high number of cases.

Only 9.7% of cases required middle and high complexity resources; these were the only cases that truly required a tertiary level hospital. In the present study, 90.3% of cases in the otorhinolaryngology emergency unit could have been solved without middle and high complexity resources, therefore not appropriate for a tertiary level hospital. The current reality increases cost, overtaxes the hospital, and is inappropriate for that level of care.

Tertiary level urgencies, those that require surgery with endoscopes or microscopes, computed tomography, magnetic resonance imaging, electroneurography, audiometry, videolaryngoscopy, nasofibroscopy, and bronchofibroscopy comprised 9.7% of cases. The main diseases in these cases were neck abscesses, complicated sinusitis, severe epistaxis, otomastoiditis and its complications, sequelae of trauma such as CSF and endolymph leaks, facial palsy, sensorineural syndromes such as peripheral facial palsy, sudden deafness, herpes zoster infection, dyspnea due to laryngeal conditions, and complicated sialadenitis.

These data show that 90.3% of cases (22,197 visits) took up time, medical teams, secretaries, nursing teams, administration and statistics personnel, and janitors, and consumed materials and medication at a tertiary level hospital. This reality demonstrates inadequacy and ineffectiveness of healthcare, requiring improved public health policies so that these non-urgent cases receive care at appropriate healthcare facilities, rather than emergency units above their level of need.

Sarmento Jr. et al.^13^ studied the problem of patients in long waiting lines for medical otorhinolaryngologic visits and surgery, and found that the most critical point that could deal with this issue is to have specialists in outpatients units. One of the principles of the unified healthcare system (SUS) - as described in the Law 8080 of 19 September 1990 - is to apply a principle of hierarchy in healthcare. This principle states that there should be four levels of healthcare: primary, secondary, tertiary, and quaternary; this is possible because of a referral/counterreferral system. Sarmento Jr. et al.^10^ consider that poor implementation of this system because of lack of funding and logistics is the cause of the problem mentioned above.

Lack of resources and logistics should not be considered the only causes of these difficulties in primary and secondary healthcare for otorhinolaryngological conditions. Lack of knowledge about basic otorhinolaryngology on the part of general practitioners is also a factor to be considered.^14^ About 25% to 40% of the medical practice of general clinicians consists of ear, nose, and throat diseases^14,^^15^. Mir et al.^15^ showed that the demand for hospital care is reduced when primary care services count on the support of an otorhinolaryngology specialist. Thus, we consider that better training of general practitioners would allow professionals in secondary level healthcare facilities to work within their specialty, which would improve the overall effectiveness of the system^14,^^16,^^17^.

The hospital admittance rate may be considered an objective parameter to assess the severity of cases. The 2.35% hospital admittance rate in our series is similar to that in other published studies - the actual percentages range from 1.4%^3^ to 6%.^6^ These papers show that this rate oscillates around 5%^2,^^7,^^18^.

We found that about 95% of cases were seen from 7 a.m. to midnight; thus, it may be inferred that this time period is overcrowded. This finding concurs with those of Pino Rivero,^11^ in which the lowest number of cases were seen from 2 a.m. to 7 a.m., and Gallo^3^, who reported that only 6% of cases were seen from midnight to 8 a.m.

The most frequent diseases were inflammatory/infectious conditions (65.71%), mostly otologic conditions, which comprised 62.72% of all inflammatory/infectious diseases. This group of diseases was the most frequent group in other papers^2,^^9,^^10,^^19,^^20^. In second place were foreign bodies - 16.77% of urgent cases (Table 2). Trauma was third (7.9%). It should be noted that the buccomaxillofacial surgery team also assists facial trauma patients, which could reduce this percentage. The paucity of pediatric patients is explained by the presence of an institution in the public hospital network that treats these patients exclusively.

Otologic complaints were the most frequent - 62.27% of cases - followed by rhinologic cases, then pharyngostomatologic patients, and head & neck surgery cases. Why do patients with otologic conditions predominate in otorhinolaryngology emergency units? A possible explanation is that otoscopy and microscopy of the nose or mouth is more difficult for physicians of other specialties. However, this was not taken into account in the present study, as the study population consisted of more than referrals only.

The frequency of otologic cases at our institution differs from that in other published studies^21,^^22^, in which pharyngeal conditions predominate.

Other studies have found a similar male and female proportion^3,^^9,^^23^; in our series, there was a 1.19 female to male ratio - there were slightly more female patients. Most of the cases were aged from 16 to 65 years. Most of the patients in this study were adults.

## CONCLUSION

The present study concluded that 61.26% of otorhinolaryngological cases seen at an otorhinolaryngological emergency unit of a tertiary level public hospital in the Federal District are urgent cases; most of these are otologic inflammatory/infectious conditions. The findings revealed that 38.73% of cases were not considered truly urgent. Among the urgent cases, only 9.7% required middle or high complexity resources, and were adequately assisted in a tertiary hospital.
